# Sexual Harassment and Verbal Violence Among Medical and Nursing Professionals: A Two-Center Cross-Sectional Study in Greece

**DOI:** 10.7759/cureus.92940

**Published:** 2025-09-22

**Authors:** Maria Papadopoulou, Polyxeni Mangoulia, Venetia-Sofia Velonaki, Athena Kalokairinou

**Affiliations:** 1 Department of Nursing, Faculty of Nursing, National and Kapodistrian University of Athens, Athens, GRC; 2 Department of Nursing, Evangelismos General Hospital, Athens, GRC

**Keywords:** healthcare professionals, nurses, physicians, sexual harassment, verbal abuse, workplace violence

## Abstract

Background: Verbal abuse is recognized as the most prevalent form of workplace violence in healthcare settings. In recent years, however, increasing attention has been paid to the issue of sexual harassment, particularly among nursing professionals.

Objective: This study aimed to examine the prevalence of verbal abuse and sexual harassment among physicians and nurses employed in public hospitals in the Attica region of Greece.

Methods: A cross-sectional survey was conducted from May to July 2023 involving 61 physicians and 279 nurses across two public hospitals. The Verbal Abuse Scale (VAS) was employed to measure verbal abuse, while the Sexual Experiences Questionnaire (SEQ) assessed experiences of sexual harassment.

Results: A total of 88.8% of participants reported experiencing verbal abuse at least once in the past year. No statistically significant difference in verbal abuse prevalence was observed between physicians and nurses. However, nurses reported significantly more frequent experiences of trivialization (p=0.011). Nurses were also more likely to endorse emotional reactions such as "I don't deserve to be treated this way" (p<0.001), "He/she has no right to treat me this way" (p=0.004), "This could potentially hurt me" (p=0.034), and "Why do I let him/her upset me enough to cry?" (p=0.004). Moreover, nurses were more likely to confront the perpetrator directly (p<0.001). Regarding sexual harassment, 70.3% of respondents indicated they had experienced such incidents at least once during their careers. Sexual harassment was significantly more prevalent among women (OR=3.356; p=0.003) and among those working in emergency departments or outpatient clinics (OR=2.696; p=0.015).

Conclusions: A considerable proportion of both medical and nursing staff are subjected to verbal abuse and sexual harassment in their workplace. These findings underscore the urgent need for robust policies and interventions by healthcare administrators and policymakers to support victims and address workplace violence without fear of retaliation.

## Introduction

Violence can affect individuals across all levels of society and may occur in various environments, including the home, public spaces, educational institutions, and workplaces [1]. The National Institute for Occupational Safety and Health defines workplace violence as "any act or threat of violence, ranging from verbal abuse to physical assaults or even homicide, that occurs in a work setting" [2]. Workplace violence is particularly prevalent in the social services sector, especially within healthcare environments [3]. Notably, healthcare professionals are reported to be 16 times more likely to experience workplace violence compared to other occupational groups [4]. In 2018 alone, healthcare workers accounted for 73% of all non-fatal violent workplace injuries and illnesses in the United States [5].

Aggressive and threatening behavior directed at healthcare professionals compromises both patient safety and the overall quality of care, creating growing concern at national and international levels [6]. The consequences of workplace violence extend beyond immediate physical harm, impacting organizational productivity, staff morale, and trust in leadership. Victims often report diminished team cohesion and perceive the work environment as unsafe and hostile [7]. Furthermore, exposure to workplace violence has been associated with increased job-related stress, absenteeism, family conflict, and higher turnover rates. Healthcare professionals who experience such incidents face an elevated risk of developing psychological disorders [8].

Among the different forms of violence in healthcare settings, verbal abuse is the most commonly reported. Healthcare professionals are three times more likely to encounter verbal abuse than physical violence [9]. A study by Silwal and Joshi reported a 36.9% prevalence of verbal abuse among nurses, with the primary perpetrators identified as patients' relatives (48.9%), staff members (23.77%), and patients (17.6%). The reported effects included intrusive memories and distressing thoughts, ultimately leading to reduced job satisfaction [10]. Another study by Toska et al. found that 38.3% of physicians and 12.4% of nurses experienced weekly verbal abuse. For physicians, accusations and blame were the most frequent forms, while nurses more often faced criticism [11].

In recent years, sexual harassment in the healthcare workplace has also received growing attention, particularly concerning nurses. Research suggests that one in four nurses has experienced sexual harassment [3], with nurses being more frequently targeted than other healthcare workers [12], likely due to the emotional and physical proximity inherent in their roles [13]. According to Papantoniou, 70% of nurses reported experiencing sexual harassment at least once in their professional careers, most commonly in the form of gender-based harassment. The primary perpetrators were male physicians. Nurses employed in private hospitals and younger nurses were found to be at greater risk. Notably, 30% of nurses did not report the incidents due to fear of retaliation or the belief that no effective action would be taken [14].

Another study found that 95.6% of 251 participating nurses and nursing students had experienced some form of sexual harassment in the past year, with verbal sexual harassment being the most common. It was particularly prevalent among younger nurses and those working in adult acute care units compared to pediatric settings. Patients were identified as the most frequent perpetrators [15]. A meta-analysis of 43 observational studies involving 52,345 nurses reported a 12.6% prevalence of sexual harassment in the past year and a 53.4% lifetime prevalence. Factors such as gender, national income level, and average age of participants were significantly associated with harassment prevalence [16].

Violence against healthcare professionals represents a global public health emergency that undermines the foundations of health systems and directly affects patient outcomes. Verbal abuse and sexual harassment not only jeopardize the physical and mental health of healthcare workers but also impair the care delivered to patients [3]. Victims of sexual harassment frequently report psychological symptoms such as anxiety, depression, sleep disturbances, weight fluctuations, reduced self-esteem, and sexual dysfunction [3]. Job satisfaction, organizational commitment, and performance may decline, while stress and burnout increase. Interpersonal conflicts among staff may also escalate. Moreover, victims often report physical symptoms such as headaches, fatigue, dizziness, gastrointestinal disturbances, menstrual irregularities, and musculoskeletal pain. Alarmingly, sexual harassment has also been linked to suicidal behavior [14,17].

Given this context, the present study aims to investigate the frequency of verbal abuse and sexual harassment among medical and nursing personnel working in public hospitals in the Attica region of Greece. The main research questions were as follows: What is the prevalence of verbal abuse and sexual harassment among medical and nursing staff? What are the main forms of verbal abuse and sexual harassment encountered? What are the effects of verbal abuse and sexual harassment on the victims? How do victims cope with incidents of verbal abuse and sexual harassment?

## Materials and methods

Study design and setting

This was a cross-sectional study conducted between May and July 2023 in two public general hospitals, namely, the Evangelismos General Hospital and Sotiria General Hospital, located in the Attica region. The first hospital had a capacity of 943 beds, and the second hospital had 710 beds. The selection of hospitals was based on convenience sampling.

Participants

The study population consisted of medical and nursing staff employed at the two selected hospitals. Inclusion criteria were as follows: (1) participants were either permanent, auxiliary, or specialist medical or nursing personnel; (2) participants had adequate proficiency in the Greek language; and (3) participation was voluntary. A total of 340 healthcare professionals took part in the study, including 61 physicians and 279 nurses. Convenience sampling was used for participant recruitment.

Data collection

Data were collected using three instruments: the Verbal Abuse Scale (VAS) [18,19], the Sexual Experiences Questionnaire (SEQ) [14,20], and a demographic and occupational characteristics form.

The VAS assesses verbal abuse in the workplace and includes 11 sections. The first six sections explore the frequency of verbal abuse incidents, the perpetrator's relationship to the victim, whether the abuse occurred in the presence of others, the stress level induced, and the perpetrator's awareness of their behavior's consequences. The remaining sections assess the frequency and severity of 10 specific verbal abuse incidents, victims' reactions, coping strategies, the severity of the consequences, and the emotional impact experienced. Responses were recorded using a five-point Likert scale [19].

Sexual harassment was assessed using the SEQ, a validated tool for examining sexual harassment in occupational settings. The questionnaire comprises two parts. Part A consists of 22 items measuring the frequency of gender harassment, unwanted sexual attention, and sexual coercion using a five-point Likert scale (1=very often; 5=never). Part B includes 16 multiple-choice items addressing the demographics of the perpetrator, the location of the harassment, and reasons for non-disclosure by victims. Additionally, two questions assessed whether victims reported the incidents and whether the hospital conducted an internal investigation [14,20]. One additional item, "Do you think you have been sexually harassed?", was included in the instrument.

The principal investigator was responsible for data collection. Questionnaires were distributed to participants during work shifts in a manner that did not interfere with the normal functioning of hospital departments. Participants were informed both verbally and in writing regarding the study's purpose, voluntary participation, data anonymity and confidentiality, and their right to withdraw at any time without consequences. The researcher remained available in each unit for approximately 30 minutes to answer questions and then returned one week later to collect the sealed questionnaires. Completed questionnaires were submitted in opaque envelopes to ensure confidentiality.

Ethical considerations

The study was conducted in accordance with the ethical principles outlined in the Declaration of Helsinki and was compliant with the General Data Protection Regulation (GDPR) 2016/679. The research protocol received ethical approval from the Ethics and Deontology Committee of the Department of Nursing at the National and Kapodistrian University of Athens (approval number: 440), as well as from the Scientific Council and the Ethics and Deontology Committee of the Evangelismos General Hospital (approval number: 134) and the Scientific Council of the Sotiria General Hospital (approval number: ΕΣ/82). Additionally, written permission was obtained from the administrative and scientific boards of the hospitals for the study to be carried out on site. Permission to use the Greek-adapted versions of the VAS and SEQ was also obtained from the respective authors. Participants were informed about the study both verbally and in writing and were asked to provide written informed consent for the anonymous use of their data for research purposes.

Data analysis

Statistical analysis was performed using IBM SPSS Statistics for Windows, V. 28.0 (IBM Corp., Armonk, NY, USA). The significance level was set at α=0.05. Descriptive statistics were used to summarize the sample characteristics. Quantitative variables were expressed as means and standard deviations (SD) or as medians and interquartile ranges (25th-75th percentiles). The normality of quantitative variables was tested using the Kolmogorov-Smirnov or Shapiro-Wilk test, as well as visual inspection of histograms.

Qualitative variables were presented as absolute numbers (N) and relative frequencies (%). Bivariate analyses were then conducted using the following tests: (a) the t-test for normally distributed quantitative variables compared with dichotomous variables, (b) the Mann-Whitney U test for non-normally distributed quantitative variables, and (c) the chi-squared test, Monte Carlo simulation, or Fisher's exact test for categorical variables.

Finally, multiple logistic regression analysis was performed using a stepwise method to identify independent predictors of sexual harassment. Odds ratios (OR), 95% confidence intervals (CI), and p-values are reported.

## Results

As shown in Table 1, 282 participants (82.9%) were women, with a mean age of 38.74 (±9.92) years. A total of 137 participants (41.4%) were married, and 120 (35.3%) held a postgraduate degree. Regarding their work department, 39% worked in a ward, 34.8% in an ICU or advanced care unit, 16.5% in an emergency department or outpatient clinic, and 9.8% in surgery/anaesthesiology. The average years of work experience was 15.01 (±10.55). Statistically significant differences were found between medical and nursing staff across various demographic variables.

**Table 1:** Demographic data

	Total sample (N=340)	Medical staff (N=61)	Nursing staff (N=279)	P-value
Gender, N (%)				
Male	58 (17.1%)	24 (39.3%)	34 (12.2%)	p<0.001
Female	282 (82.9%)	37 (60.7%)	245 (87.8%)	
Age (years), mean (±SD)	38.74 (±9.92)	34.91 (±9.12)	39.78 (±9.89)	p=0.004
Education level, N (%)				
Secondary education	71 (20.9%)	0 (0%)	71 (24.5%)	p<0.001
Technological education	108 (31.8%)	0 (0%)	107 (38.4%)	
University education	41 (12.1%)	18 (29.5%)	24 (8.6%)	
M.Sc./Ph.D.	120 (35.3%)	43 (70.5%)	77 (27.6%)	
Marital status, N (%)				
Unmarried	156 (47.1%)	35 (59.3%)	121 (44.5%)	p=0.117
Married	137 (41.4%)	19 (32.2%)	118 (43.3%)	
Work experience (years), mean (±SD)	15.01 (±10.55)	9.88 (±9.09)	16.14 (±10.53)	p<0.001
Work setting, N (%)				
Surgery/anaesthesiology	32 (9.8%)	6 (10.7%)	26 (9.6%)	-
Emergency department/outpatient clinic	54 (16.5%)	4 (7.1%)	50 (18.4%)	-
ICU/advanced care unit	114 (34.8%)	14 (25%)	100 (36.8%)	-

Verbal abuse

A total of 302 participants (88.8%) reported having experienced verbal abuse at least once in the past year, while 22.05% reported incidents at least once per week. The frequency of verbal abuse did not differ significantly between physicians and nurses (93.4% vs. 87.8%; p=0.206). Among those affected, 81.5% of physicians and 77.9% of nurses stated that the abuse occurred in the presence of others (p=0.339). Regarding the perpetrator, physicians most often identified a senior staff member or a patient's relative or the patient themselves. Nurses most commonly identified the patient's relative (38.7%), the patient (33.3%), and a senior staff member (27.6%) (Appendix 1).

Appendix 2 presents the frequency and intensity of different verbal abuse behaviors. The most frequent behaviors were judging and criticizing (3.23 (±1.37)), accusing and blaming (3.17 (±1.36)), and abuse disguised as a joke (3.01 (±1.35)). Nurses reported significantly more experiences of trivializing (p=0.011). The most stressful verbal abuse behaviors were accusing and blaming (3.26 (±1.53)), judging and criticizing (3.21 (±1.43)), and trivializing (3.03 (±1.53)).

Common thoughts following verbal abuse included "I don't deserve to be treated this way" (3.53 (±1.50)), "He/she has no right to treat me this way" (3.63 (±1.52)), and "I haven't done anything wrong" (3.17 (±1.63)). Nurses were more likely to report thoughts of injustice, harm, and emotional upset (p<0.05) (Appendix 3).

Appendix 4 shows the coping strategies used by professionals after verbal abuse. Most frequently, participants tried to clarify misunderstandings (3.17 (±1.31)) or detached themselves from the situation (2.73 (±1.41)). Nurses were significantly more likely than physicians to directly confront the perpetrator (p<0.001).

The most common effects of verbal abuse (Appendix 5) included reduced job satisfaction (2.56 (±1.39)), deterioration of colleague relationships (2.51 (±1.28)), and reduced workplace well-being (2.48 (±1.32)). Nurses more frequently reported a decrease in trust and support in the workplace (p=0.039).

Emotional responses to verbal abuse (Appendix 6) included anger (3.39 (±1.31)), sadness (2.83 (±1.45)), disgust (2.79 (±1.48)), and frustration (2.74 (±1.33)). Nurses more often reported embarrassment/humiliation (p=0.002) and discomposure (p=0.028).

Sexual harassment

Of the 340 participants, 151 (44.4%) self-reported experiencing sexual harassment, while 239 (70.3%) reported incidents when completing the SEQ. The frequency of harassment did not significantly differ between physicians and nurses (65.6% vs. 71.3%; p=0.439) (Table 2).

**Table 2:** Frequency of sexual harassment behaviors

Sexual harassment behaviors	Total sample (N=340)	Medical staff (N=61)	Nursing staff (N=279)	P-value
	Mean (±SD)	Mean (±SD)	Mean (±SD)	
Gender harassment				
Has treated you differently because of your sex	2.11 (±1.23)	2.35 (±1.45)	2.06 (±1.18)	p=0.163
Displayed, used, or distributed sexist or suggestive materials	1.43 (±0.86)	1.62 (±0.95)	1.38 (±0.83)	p=0.073
Made offensive sexist remarks	1.79 (±1.10)	2.01 (±1.29)	1.74 (±1.05)	p=0.131
Put you down or was condescending to you because of your sex	1.83 (±1.13)	1.98 (±1.29)	1.79 (±1.09)	p=0.296
Unwanted sexual attention				
Repeatedly told sexual stories or jokes that were offensive to you	1.68 (±1.02)	1.73 (±1.07)	1.67 (±1.00)	p=0.622
Whistled, called, or hooted at you in a sexual way	1.53 (±0.93)	1.55 (±0.97)	1.53 (±0.92)	p=0.860
Made unwelcome attempts to draw you into a discussion of sexual matters	1.49 (±0.88)	1.55 (±0.94)	1.48 (±0.87)	p=0.519
Made crude and offensive sexual remarks, either publicly or privately	1.62 (±0.97)	1.72 (±1.05)	1.59 (±0.94)	p=0.356
Made offensive remarks about your appearance, body, or sexual activities	1.57 (±0.94)	1.61 (±0.98)	1.55 (±0.93)	p=0.721
Made gestures or used body language of a sexual nature which embarrassed or offended you	1.47 (±0.86)	1.50 (±0.91)	1.46 (±0.85)	p=0.728
Stared, leered, or ogled at you in a way that made you feel uncomfortable	1.67 (±1.05)	1.61 (±1.00)	1.69 (±1.06)	p=0.567
Has he made unwanted attempts to establish a romantic sexual relationship with you despite your efforts to discourage it?	1.31 (±0.73)	1.18 (±0.47)	1.34 (±0.77)	p=0.035
Continued to ask you for dates, drinks, dinner, and so on, even though you said "No"	1.35 (±0.79)	1.18 (±0.59)	1.39 (±0.83)	p=0.022
Touched you in a way that made you feel uncomfortable	1.42 (±0.83)	1.42 (±0.90)	1.41 (±0.81)	p=0.953
Sexual coercion				
Made unwanted attempts to stroke, fondle, or kiss you	1.23 (±0.62)	1.34 (±0.91)	1.20 (±0.54)	p=0.251
Attempted to have sex with you without your consent or against your will, but was unsuccessful	1.12 (±0.49)	1.08 (±0.42)	1.12 (±0.49)	p=0.494
Had sex with you without your consent or against your will	1.09 (±0.46)	1.04 (±0.28)	1.10 (±0.49)	p=0.405
It made you feel like you were being bribed with some reward or special treatment to engage in sexual behavior	1.19 (±0.61)	1.26 (±0.70)	1.18 (±0.58)	p=0.357
It made you feel threatened with some retaliation for not being sexually cooperative	1.13 (±0.55)	1.06 (±0.31)	1.14 (±0.58)	p=0.292
Treated you badly for refusing to have sex	1.17 (±0.64)	1.08 (±0.27)	1.19 (±0.69)	p=0.036
Implied faster promotions or better treatment if you were sexually cooperative	1.18 (±0.65)	1.18 (±0.53)	1.18 (±0.67)	p=0.947
It made you afraid you would be treated poorly if you didn't cooperate sexually	1.14 (±0.58)	1.08 (±0.33)	1.16 (±0.63)	p=0.339

The most frequent behaviors were being treated differently because of sex (2.11 (±1.23)), offensive sexist remarks (1.79 (±1.10)), sexist comments, offensive jokes, and crude sexual remarks.

Significantly more nurses than physicians reported behaviors such as unwanted romantic advances (p=0.035), repeated invitations despite refusal (p=0.022), and retaliation for refusing sex (p=0.036). Men reported significantly fewer incidents of sexual harassment compared to women (27.6% vs. 47.9%; p=0.005). Harassment prevalence also varied significantly by department (p=0.028), being highest in emergency department/outpatient clinics (54.5%) and ICUs (48.7%) (Table 3).

**Table 3:** Comparison of demographic and professional characteristics of the sample regarding the experience of sexual harassment *Values are presented as mean (±SD).

Demographic characteristics	Sexual harassment				P-value
	No		Yes		
	Ν	%	Ν	%	
Gender					
Male	42	72.4	16	27.6	p=0.005
Female	148	52.1	136	47.9	
Age (years)*	39.53 (±10.60)		37.82 (±9.00)		p=0.217
Education level					
Secondary education	40	21.2	31	20.5	p=0.382
Technological education	62	32.8	45	29.8	
University education	27	14.3	15	9.9	
M.Sc./Ph.D.	60	31.7	60	39.7	
Marital status					
Unmarried	89	57.1	67	42.9	p=0.090
Married	81	59.1	56	40.9	
Divorced	15	39.5	23	60.5	
Department of the hospital					
Ward	77	60.2	51	39.8	p=0.028
Surgery/anaesthesiology	24	75	8	25	
Emergency department/outpatient clinic	24	45.5	30	54.5	
ICU/advanced care unit	59	51.3	56	48.7	
Work experience (years)*	15.99 (±10.73)		13.72 (±10.21)		p=0.064

Table 4 presents the logistic regression results: women were 3.356 times more likely than men to report sexual harassment (p=0.003), and staff in emergency departments were 2.696 times more likely than those in wards to report sexual harassment (p=0.015).

**Table 4:** Results of multiple logistic regression with frequency of sexual harassment as the dependent variable

	OR	95% CI	P-value
Gender			
Male	Reference		
Female	3.356	1.517-7.424	0.003
Department of the hospital			
Ward	Reference		
Surgery/anaesthesiology	0.627	0.237-1.663	0.349
Emergency department/outpatient clinic	2.696	1.216-5.977	0.015
ICU/advanced care unit	1.646	0.901-3.007	0.105

Characteristics and effects of sexual harassment

Perpetrators were most commonly male doctors (48.3%), followed by patients (28.5%), relatives of patients (20.5%), and male nurses (17.9%). Over half (53%) of victims reported the perpetrator had a higher status. The harassment occurred mostly in patient care units (37.7%), corridors (34.4%), offices (24.5%), or exam areas (22.5%).

Common reactions to harassment included ignoring the behavior (52%), confronting the perpetrator (40.1%), not reacting or reporting (38.2%), and discussing it with friends/family (33.6%).

Emotional responses included feeling uncomfortable (52%), embarrassed (47.4%), angry (43.4%), frustrated (36.2%), and nervous (34.2%). About 38.4% of victims experienced physical effects, such as headache (43.1%), insomnia (37.9%), and stomachache (34.5%). Also, 46.7% experienced mental health issues, most commonly anxiety and low mood (62.9%), low self-confidence (37.1%), and guilt (30%).

Work performance was negatively affected in 43.8% of victims, most often due to avoiding coworkers (54.5%), low job satisfaction (53%), less contact with others (40.9%), and difficulty concentrating (36.4%).

Only 12 participants (7.9%) reported the incident to Human Resources or a supervisor. Of those, six (50%) reported a formal investigation. Reasons for non-reporting included the following: belief that nothing would happen (44.7%), fear of being seen as overreacting (30.7%), fear of disbelief (21.1%), concerns over confidentiality (22.1%), and fear of harming workplace relationships (20%).

## Discussion

Globally, professionals, particularly nurses, are affected by sexual harassment in the workplace [16]. Sexual harassment refers to any sexually or gender-based behavior that both is unwanted and violates the dignity of the individual [15]. At the same time, verbal abuse is a common form of workplace violence in healthcare organizations. It has been argued that verbal abuse is a less extreme but more prevalent type of violence that has received less attention than physical assaults [21]. This study aimed to investigate the prevalence of verbal abuse and sexual harassment among healthcare professionals.

One of the main findings was that seven out of 10 healthcare professionals had experienced at least one act of sexual harassment. The five most frequently reported behaviors included being treated differently due to gender, being spoken to condescendingly because of gender, hearing offensive or sexist comments, being subjected to repeated sexual jokes or stories, and receiving inappropriate or offensive sexual comments. These behaviors point to a predominance of gender-based and verbal sexual harassment. Similar findings were reported in the study by Papantoniou, where 70% of nurses had experienced sexual harassment at least once in their career, most commonly gender harassment [14]. Another study reported that 95.6% of participants experienced some form of sexual harassment in the past 12 months, with verbal sexual harassment being the most frequent [15].

The prevalence and perpetrators of sexual harassment vary depending on the country, region, culture, and healthcare setting. For example, in the Global North (e.g., Switzerland), patients are more commonly identified as perpetrators, while in the Global South, perpetrators may include colleagues, relatives, or physicians. Although sexual harassment is increasingly recognized as an occupational health concern, it often remains unreported. Many healthcare professionals choose not to report incidents, regardless of who is responsible [3].

Our study also found that sexual harassment rates were similar between physicians and nurses. Generally, nurses are at higher risk for sexual harassment due to the nature of their work, which often involves physical contact and interactions with individuals under stress or the influence of substances [22].

Moreover, women were 3.356 times more likely than men to report sexual harassment. In Papantoniou's study, 67% of female nurses had experienced sexual harassment, compared to 41% of male nurses [17]. Healthcare workers in emergency departments and outpatient clinics were 2.696 times more likely to report harassment compared to those in other departments. Previous research has shown that sexual harassment is more common among younger and single nurses [15,23].

In this study, perpetrators were most often male physicians, patients, patients' companions, and male nurses. Similarly, Papantoniou et al. found male doctors to be the primary perpetrators [14]. Another study with male nurses as participants reported that fellow nurses and patients were the most common perpetrators [24]. Kahsay et al. found that perpetrators included patients (46.59%), families (27.74%), medical staff (41.12%), nurses (20%), and other colleagues (17.8%) [3].

As for how victims reacted, about half ignored the behavior or avoided the perpetrator, while four in 10 responded directly. Other reactions included staying silent, discussing the incident with friends or colleagues, or formally reporting it. In one study, responses included laughing it off (32.3%), saying nothing (12.5%), or avoiding the situation (9.4%) [25].

Additionally, four out of 10 nurses reported experiencing physical or mental health issues following harassment, and 43.6% reported reduced job performance. Prior research confirms these effects, showing high rates of psychological and physical consequences such as stress, anxiety, gastrointestinal symptoms, and exhaustion [12,22,25]. One review found that 42.8% of nurses developed anxiety or depression, while 30.19% experienced physical symptoms, and over 61% reported emotional distress [3].

In our study, only 7.9% of professionals reported incidents to Human Resources or supervisors. Reasons for not reporting included beliefs that nothing would change, fear of being accused of overreacting, concerns over credibility, and lack of confidentiality. Similarly, Papantoniou found that 30% of nurses refrained from reporting due to fear of consequences or disbelief [14].

There is a noted absence of guidelines and interventions addressing workplace sexual harassment [15]. Moreover, there are a few educational initiatives for nurses. According to Nielsen et al. and Kim et al., such education could help nurses cope with harassment and reduce feelings of depression or insecurity [22,26].

Regarding verbal abuse, 90% of healthcare professionals reported experiencing it at least once per year. No significant differences were found between physicians and nurses. Similar findings were reported in Greek studies, where verbal abuse occurred weekly among nurses [21]. Giannakouli found that 79.4% of physicians and 91.7% of nurses had experienced verbal abuse [21]. Toska et al. found weekly verbal abuse in 38.3% of doctors and 12.4% of nurses [11].

Common forms of verbal abuse included judging and criticizing, blaming and accusing, and abuse masked as jokes. Nurses experienced trivializing more frequently than physicians. Similar patterns were observed in Ergazaki's study in Crete, where the primary behaviors reported were intense criticism and blame [27]. Toska et al. also noted that accusation and blaming were most common among physicians, while criticism predominated among nurses [11].

In terms of perpetrators, they included patients' relatives, patients, non-supervisory senior staff, and immediate supervisors. Silwal and Joshi found relatives to be the most frequent perpetrators (48.9%), followed by staff (23.77%) and patients (17.6%) [10]. Toska et al. reported similar findings: 26.7% of physicians and 31.9% of nurses identified another senior staff member as the abuser [11].

Verbal abuse had notable consequences, including reduced job satisfaction, strained colleague relationships, decreased workplace comfort, mental health impacts, and diminished trust and support. Silwal and Joshi reported that disturbing memories and reduced job satisfaction were key outcomes [10]. Toska et al. and Giannakouli also reported that verbal abuse negatively impacted teamwork and care quality [11,21].

Nurses cannot provide effective care during or after episodes of violence. Verbal abuse disrupts the care process and contributes to missed nursing care (MNC), defined as necessary care that is omitted, delayed, or incomplete. MNC is considered an indicator of patient safety and care quality [28]. In one study, the most common types of verbal violence were cursing, insults, shouting, and threats. Reactions included ignoring the abuse, adapting, or wishing to escape. Lost care involved non-therapeutic communication, delayed care, or withdrawal. Ultimately, verbal abuse negatively affected nurse-patient interactions and contributed to missed care [29].

Practical strategies to address verbal abuse and sexual harassment in healthcare settings include the implementation of clear institutional zero-tolerance policies, regular staff education and training programs, and the establishment of confidential and accessible reporting mechanisms. Strengthening organizational support systems and promoting a culture of respect and accountability can further empower healthcare professionals to report incidents and reduce the prevalence of workplace harassment. Such measures are essential for safeguarding the mental health and job performance of staff while also ensuring patient safety and the quality of care.

Despite the important findings of this study, several limitations should be noted. First, the selection of hospitals and participants was based on convenience sampling. The sample included mostly female nurses, with limited representation from male staff and physicians. Second, the questionnaire was lengthy, and participants completed it during work shifts, which may have limited their attention due to time constraints.

## Conclusions

The present study revealed that nine out of 10 healthcare professionals had experienced verbal abuse, while seven out of 10 had encountered sexual harassment in the workplace. The prevalence of both forms of violence was similar among physicians and nurses. Female professionals and those working in emergency departments or outpatient clinics were more likely to report incidents of sexual harassment. Most victims adopted a passive response, with only a small percentage formally reporting the incidents to Human Resources or other responsible authorities. Such acts of violence were found to significantly affect victims' physical and mental health, as well as their job performance.

These findings underscore the urgent need for increased awareness, formal recognition, and the implementation of clear and structured interventions to prevent and address workplace violence in healthcare settings. Protecting the well-being of healthcare workers, particularly those on the front lines, is essential to ensure the effective, safe, and continuous delivery of healthcare services to the wider community.

Future research should focus on multi-center and longitudinal studies to provide more generalizable evidence, as well as interventional studies evaluating the effectiveness of preventive and supportive strategies. Such approaches will help avoid duplicate studies and offer practical guidance for reducing workplace harassment in healthcare settings.

## Appendices

Appendix 1

**Table 5:** Abuser characteristics

	Total sample	Physicians	Nurses	P-value
	N (%)	N (%)	N (%)	
Did the episode of verbal abuse take place in the presence of others?				
No	62 (21.5)	10 (18.5)	52 (22.1)	p=0.339
Yes	227 (78.5)	44 (81.5)	183 (77.9)	
Relationship between the victim of verbal abuse and the abuser				
Direct supervisor	62 (20.8)	12 (21.8)	50 (20.6)	p=0.838
Other senior member	85 (28.5)	18 (32.7)	67 (27.6)	p=0.445
Direct subordinate	33 (11.1)	3 (5.5)	30 (12.3)	p=0.141
Other subordinate	14 (4.7)	13 (5.3)	1 (1.8)	p=0.326
Relative of the patient	112 (37.6)	18 (32.7)	94 (38.7)	p=0.410
Patient	99 (33.2)	18 (32.7)	81 (33.3)	p=0.931
Visitor	51 (17.1)	10 (18.2)	41 (16.9)	p=0.816
Colleague	30 (10.1)	27 (11.1)	3 (5.5)	p=0.208
Level of stress resulting from verbal abuse				
Not at all	5 (1.7)	3 (5.3)	2 (0.8)	p=0.068
Almost no	17 (5.6)	5 (8.8)	12 (4.9)	
A little	29 (9.6)	6 (10.5)	23 (9.4)	
High enough	84 (27.8)	16 (28.1)	68 (27.8)	
Very high	55 (18.2)	13 (22.8)	42 (17.1)	
Too high	73 (24.2)	7 (12.3)	66 (26.9)	
Extremely high	39 (12.9)	7 (12.3)	32 (13.1)	

Appendix 2

**Table 6:** Frequency and intensity of verbal abuse forms between physicians and nurses (mean, standard deviation, and significance between groups)

Verbal abuse forms	Total sample	Physicians	Nurses	P-value
	Mean (±SD)	Mean (±SD)	Mean (±SD)	
	Frequency			
Abusive anger	2.39 (±1.37)	2.46 (±1.36)	2.37 (±1.37)	p=0.675
Condescending	2.24 (±1.18)	2.30 (±1.19)	2.23 (±1.18)	p=0.683
Abuse disguised as a joke	3.01 (±1.35)	2.91 (±1.38)	3.03 (±1.34)	p=0.530
Ignoring	2.71 (±1.38)	2.69 (±1.43)	2.71 (±1.37)	p=0.938
Trivializing	2.69 (±1.44)	2.22 (±1.31)	2.79 (±1.45)	p=0.011
Accusing and blaming	3.17 (±1.36)	2.92 (±1.13)	3.22 (±1.40)	p=0.156
Judging and criticizing	3.23 (±1.37)	3.26 (±.1.24)	3.22 (±1.40)	p=0.828
Discounting	2.63 (±1.37)	2.42 (±1.36)	2.67 (±1.37)	p=0.237
Threatening	2.19 (±1.36)	2.00 (±1.22)	2.24 (±1.39)	p=0.260
Sexual harassment	1.73 (±1.21)	1.93 (±1.35)	1.67 (±1.17)	p=0.198
	Stressfulness			
Abusive anger	2.87 (±1.46)	3.02 (±1.31)	2.83 (±1.49)	p=0.438
Condescending	2.12 (±1.15)	2.26 (±1.12)	2.09 (±1.15)	p=0.373
Abuse disguised as a joke	2.82 (±1.33)	3.00 (±1.41)	2.77 (±1.30)	p=0.278
Ignoring	2.60 (±1.41)	2.44 (±1.40)	2.64 (±1.41)	p=0.364
Trivializing	3.03 (±1.53)	2.88 (±1.52)	3.06 (±1.54)	p=0.448
Accusing and blaming	3.26 (±1.53)	3.25 (±1.36)	3.25 (±1.46)	p=0.954
Judging and criticizing	3.21 (±1.43)	3.32 (±1.36)	3.19 (±1.45)	p=0.559
Discounting	2.84 (±1.46)	2.79 (±1.39)	2.86 (±1.48)	p=0.772
Threatening	2.62 (±1.57)	2.51 (±1.47)	2.64 (±1.59)	p=0.595
Sexual harassment	1.98 (±1.44)	2.15 (±1.46)	1.94 (±1.43)	p=0.378

Appendix 3

**Table 7:** First thoughts after the verbal abuse incident (mean, standard deviation, and significance between groups)

First thoughts	Frequency			
	Total sample	Physicians	Nurses	P-value
	Mean (±SD)	Mean (±SD)	Mean (±SD)	
What a jerk	2.11 (±1.40)	2.26 (±1.51)	2.08 (±1.38)	p=0.369
I don't deserve to be treated this way	3.53 (±1.50)	2.89 (±1.58)	3.69 (±1.44)	p<0.001
Ι can deal with this	2.49 (±1.39)	2.77 (±1.55)	2.42 (±1.34)	p=0.092
He/she has no right to treat me this way	3.63 (±1.52)	3.10 (±1.57)	3.75 (±1.48)	p=0.004
I haven't done anything wrong	3.17 (±1.63)	3.32 (±1.50)	3.15 (±1.66)	p=0.484
I am going to be in trouble over this	1.85 (±1.21)	1.68 (±1.00)	1.88 (±1.24)	p=0.248
Why can't I do something right when I work with him/her?	1.76 (±1.15)	1.96 (±1.37)	1.72 (±1.08)	p=0.149
It doesn't matter	2.16 (±1.21)	2.23 (±1.26)	2.15 (±1.20)	p=0.639
I can't handle this	1.93 (±1.20)	1.78 (±0.98)	1.96 (±1.25)	p=0.317
This could potentially hurt me	2.09 (±1.23)	1.82 (±0.98)	2.16 (±1.28)	p=0.034
It must be my fault	1.56 (±0.93)	1.61 (±0.88)	1.56 (±1.28)	p=0.679
I have never had a problem with anyone else	2.18 (±1.38)	2.17 (±1.32)	2.18 (±1.39)	p=0.965
Why am I always the one who causes him/her a problem? He/she is not shouting at anyone else	1.71 (±1.16)	1.72 (±1.01)	1.70 (±1.19)	p=0.933
Why do I let him/her upset me enough to cry?	1.90 (±1.37)	1.52 (±0.94)	1.98 (±1.44)	p=0.004

Appendix 4

**Table 8:** Coping behaviors and their perceived effectiveness (mean, standard deviation, and significance between groups)

Coping behavior	Frequency			
	Total sample	Physicians	Nurses	P-value
	Mean (±SD)	Mean (±SD)	Mean (±SD)	
I try to put the situation in perspective	2.43 (±1.28)	2.59 (±1.33)	2.39 (±1.27)	p=0.274
I ask for assistance/support from others	2.32 (±1.31)	2.40 (±1.39)	2.31 (±1.29)	p=0.636
I attempt to clarify any misunderstanding	3.17 (±1.31)	3.21 (±1.42)	3.15 (±1.28)	p=0.793
I engage in positive activities that directly reduce my tension	2.55 (±1.29)	2.40 (±1.31)	2.59 (±1.29)	p=0.329
I talk to myself in a reassuring way	2.47 (±1.33)	2.54 (±1.33)	2.45 (±1.34)	p=0.667
I deal directly with the abuser about the abuse	2.15 (±1.34)	1.68 (±1.07)	2.27 (±1.37)	p<0.001
I become silent with the abuser	1.99 (±1.24)	1.93 (±1.13)	2.00 (±1.27)	p=0.670
I detach myself from the situation	2.73 (±1.41)	2.65 (±1.36)	2.75 (±1.42)	p=0.627
I try to keep my feelings to myself	2.52 (±1.36)	2.54 (±1.29)	2.51 (±1.39)	p=0.876
I engage in controversial activities that reduce my tension	2.09 (±1.17)	1.96 (±1.10)	2.12 (±1.19)	p=0.361
I tend to blame myself	1.49 (±0.87)	1.65 (±0.95)	1.45 (±0.85)	p=0.131
I engage in wishful thinking	2.19 (±1.19)	2.05 (±1.12)	2.22 (±1.21)	p=0.327
I try to deal with humor	2.33 (±1.31)	2.35 (±1.29)	2.33 (±1.32)	p=0.922

Appendix 5

**Table 9:** Effects of verbal abuse (mean, standard deviation, and significance between groups)

Effects of verbal abuse	Frequency			
	Total sample	Physicians	Nurses	P-value
	Mean (±SD)	Mean (±SD)	Mean (±SD)	
Deterioration of the relationship with the colleague	2.51 (±1.28)	2.63 (±1.34)	2.48 (±1.25)	p=0.439
Deteriorating relationships with people outside the work environment	1.83 (±1.13)	1.89 (±1.16)	1.81 (±1.13)	p=0.623
Deterioration of the relationship with the rest of the staff	1.63 (±0.95)	1.57 (±0.94)	1.65 (±0.95)	p=0.595
Decreased trust and support in the workplace	2.11 (±1.24)	1.84 (±1.06)	2.18 (±1.27)	p=0.039
Decreased self-esteem	1.71 (±1.07)	1.59 (±0.94)	1.74 (±1.10)	p=0.355
Decreased self-confidence	1.63 (±1.02)	1.63 (±0.92)	1.64 (±1.04)	p=0.951
Impact on mental health	2.22 (±1.28)	2.07 (±1.17)	2.24 (±1.30)	p=0.345
Reduced performance and compliance with professional requirements	1.07 (±1.02)	1.63 (±0.92)	1.72 (±1.04)	p=0.562
Health burden	2.03 (±1.25)	1.85 (±1.18)	2.06 (±1.27)	p=0.265
Reduce the feeling of comfort/well-being in the workplace	2.48 (±1.32)	2.59 (±1.32)	2.46 (±1.32)	p=0.474
Reduced job satisfaction	2.56 (±1.39)	2.65 (±1.32)	2.53 (±1.41)	p=0.591
Increase in hours of absence from work	1.51 (±0.97)	1.72 (±1.22)	1.46 (±0.90)	p=0.071
Protest of other employees as they shoulder the abstention of the verbally abused colleague	1.57 (±0.91)	1.52 (±0.82)	1.58 (±0.93)	p=0.649

Appendix 6

**Table 10:** Emotional reactions after the verbal abuse incident (mean, standard deviation, and significance between groups)

Emotional reaction	Frequency			
	Total sample	Physicians	Nurses	P-value
	Mean (±SD)	Mean (±SD)	Mean (±SD)	
Frustration	2.74 (±1.33)	2.63 (±1.37)	2.75 (±1.31)	p=0.514
Anger	3.39 (±1.31)	3.15 (±1.37)	3.44 (±1.29)	p=0.143
Disgust	2.79 (±1.48)	2.51 (±1.42)	2.86 (±1.49)	p=0.110
Embarrassment or humiliation	2.26 (±1.36)	1.82 (±1.08)	2.36 (±1.41)	p=0.002
Sadness	2.83 (±1.45)	2.59 (±1.36)	2.86 (±1.46)	p=0.175
Helpless	1.75 (±1.15)	1.75 (±1.13)	1.74 (±1.14)	P=0.965
Powerless	1.82 (±1.22)	1.64 (±1.08)	1.86 (±1.25)	p=0.229
Shock and surprise	2.63 (±1.39)	2.37 (±1.37)	2.69 (±1.40)	p=0.118
Confused	2.15 (±1.25)	2.05 (±1.19)	2.18 (±1.27)	p=0.505
Responsible	1.67 (±1.12)	1.54 (±0.96)	1.71 (±1.15)	p=0.313
Threatened	2.08 (±1.32)	1.96 (±1.16)	2.11 (±1.35)	p=0.454
Discomposure	2.46 (±1.41)	2.12 (±1.21)	2.53 (±1.44)	p=0.028
Defeated	1.57 (±0.97)	1.65 (±1.21)	1.55 (±0.91)	p=0.495
Indifferent	1.88 (±1.12)	1.93 (±1.21)	1.86 (±1.10)	p=0.696
Feared	1.72 (±0.99)	1.74 (±0.99)	1.71 (±1.00)	p=0.900
Isolated	1.68 (±1.08)	1.65 (±1.01)	1.69 (±1.11)	p=0.800
Misunderstood	2.39 (±1.33)	2.45 (±1.37)	2.38 (±1.33)	p=0.713
Not supported	2.23 (±1.32)	2.19 (±1.30)	2.24 (±1.33)	p=0.806
